# Supplementary material for: An open-source framework for large-scale, flexible evaluation of biomedical text mining systems
Source: J Biomed Discov Collab. 2008 Jan 29;3:1. doi: 10.1186/1747-5333-3-1 (PMC2276192; doi:10.1186/1747-5333-3-1)
Supplement: Additional file 1 — Evaluation platform source code. The additional file contains the software evaluation framework discussed in this paper. The most current release can be downloaded from SourceForge.net [1]. See the accompanying README file for instructions on installation and use. Also included in the distribution is a collection of UIMA wrappers for some commonly used BioNLP tools and annotated corpora. [file 1747-5333-3-1-S1.zip › bionlp_uima_repository-1.0.1/docs/api/src-html/edu/uchsc/ccp/util/xml/XMLValidityChecker.html]

```
001    package edu.uchsc.ccp.util.xml;
002    
003    import java.io.IOException;
004    
005    import org.xml.sax.SAXException;
006    import org.xml.sax.SAXParseException;
007    import org.xml.sax.XMLReader;
008    import org.xml.sax.helpers.XMLReaderFactory;
009    
010    /**
011     * This class utilizes the XMLReader class to validate XML.
012     * <p>
013     * The code in this class was modified from: http://www.cafeconleche.org/books/xmljava/chapters/ch09s06.html
014     * 
015     * @author William A Baumgartner, Jr.
016     * 
017     */
018    public class XMLValidityChecker {
019    
020        /**
021         * Validates the input XML file.
022         * 
023         * @param xmlFile
024         *            XML file to be validated
025         * @return true is XML is valid, false otherwise
026         */
027        public static boolean validateXML(String xmlFile) {
028    
029            try {
030                XMLReader parser = XMLReaderFactory.createXMLReader();
031                parser.parse(xmlFile);
032                /* xml file is well-formed so return true */
033                return true;
034            } catch (SAXParseException e) {
035                System.err.print("ERROR -- XMLValidityChecker: " + xmlFile + " is not well-formed at Line " + e.getLineNumber() + ", column "
036                        + e.getColumnNumber() + " in the entity " + e.getSystemId());
037                return false;
038            } catch (SAXException e) {
039                System.err.println("ERROR -- XMLValidityChecker: Could not check document because " + e.getMessage());
040                return false;
041            } catch (IOException e) {
042                System.out.println("ERROR -- XMLValidityChecker: Due to an IOException, the parser could not check " + xmlFile);
043                e.printStackTrace();
044                return false;
045            }
046    
047        }
048    
049    }
```
